# An Integrated Bioinformatic Analysis of the S100 Gene Family for the Prognosis of Colorectal Cancer

**DOI:** 10.1155/2020/4746929

**Published:** 2020-11-26

**Authors:** Meng-Lu Zeng, Xian-Jin Zhu, Jin Liu, Peng-Chong Shi, Yan-Li Kang, Zhen Lin, Ying-Ping Cao

**Affiliations:** ^1^Department of Clinical Laboratory Medicine, Fujian Medical University Union Hospital, Fuzhou 350000, China; ^2^Department of Clinical Laboratory Medicine, Fujian Provincial Hospital, Fuzhou 350001, China

## Abstract

**Background:**

S100 family genes exclusively encode at least 20 calcium-binding proteins, which possess a wide spectrum of intracellular and extracellular functions in vertebrates. Multiple lines of evidences suggest that dysregulated S100 proteins are associated with human malignancies including colorectal cancer (CRC). However, the diverse expression patterns and prognostic roles of distinct S100 genes in CRC have not been fully elucidated.

**Methods:**

In the current study, we analyzed the mRNA expression levels of S100 family genes and proteins and their associations with the survival of CRC patients using the Oncomine analysis and GEPIA databases. Expressions and mutations of S100 family genes were analyzed using the cBioPortal, and protein-protein interaction (PPI) networks of S100 proteins and their mutation-related coexpressed genes were analyzed using STRING and Cytoscape.

**Results:**

We observed that the mRNA expression levels of S100A2, S100A3, S100A9, S100A11, and S100P were higher and the level of S100B was lower in CRC tissues than those in normal colon mucosa. A high S100A10 levels was associated with advanced-stage CRC. Results from GEPIA database showed that highly expressed S100A1 was correlated with worse overall survival (OS) and disease-free survival (DFS) and that overexpressions of S100A2 and S100A11 were associated with poor DFS of CRC, indicating that S100A1, S100A2, and S100A11 are potential prognostic markers. Unexpectedly, most of S100 family genes showed no significant prognostic values in CRC.

**Conclusions:**

Our findings, though still need to be ascertained, offer novel insights into the prognostic implications of the S100 family in CRC and will inspire more clinical trials to explore potential S100-targeted inhibitors for the treatment of CRC.

## 1. Introduction

The S100 family, with a common Ca ^2+^-binding motif, EF-hand, contains a group of low molecular weight acidic polypeptides (*M*_*r*_ between 9 and 14 kDa) [1], of which more than 20 S100 proteins are encoded in the human genome [2]. The S100 proteins appear to be involved in a multitude of biological processes, including calcium homeostasis, cell growth, invasion and motility, apoptosis, protein phosphorylation, chemotaxis, and inflammation [1–3]. Extensive evidence suggests that the deregulated expression of S100 proteins is closely linked to tumor progression and drug resistance in the treatment of many malignant tumors, including ovarian cancer [4], breast cancer [5], prostate cancer [6], and colorectal cancer [7].

Colorectal cancer (CRC), with high morbidity and mortality, is one of the most common malignant cancers of the digestive tract worldwide [8]. The prevalence of CRC has gradually increased owing to environmental deterioration and unhealthy lifestyle, as well as the contribution of new diagnostic techniques [9]. Despite considerable improvements in the diagnosis and treatment of CRC, many patients are diagnosed at advanced stages or relapse, which is associated with a poor prognosis for survival. A previous study showed that the 5-year overall survival (OS) rate of patients with metastatic colorectal cancer (mCRC) remains less than 15% [10]. Hence, identifying biomarkers for diagnosis and prognosis of CRC is the first imperative for developing valuable prognostic markers and individualized therapeutics.

Some relevant literatures report abnormal expressions of the S100 genes and their associations with clinicopathological characteristics and prognosis in human CRC. To the best of the authors' knowledge, the roles of S100s in CRC have not yet been explored using bioinformatics analysis. Integrating the online high-throughput microarray analysis of gene expression and copy number variants (CNVs) from massive platform data, we exhaustively analyzed the expressions and mutations of various S100 genes to determine the distinct expression patterns, numerous functions, and potential prognostic value of S100s in CRC.

## 2. Materials and Methods

### 2.1. Oncomine Analysis

Oncomine (http://www.oncomine.org), an online gene expression array database and web-based data-mining platform containing 715 datasets and 86733 samples, is frequently used to stimulate discovery in genome-wide expression analyses. Here, the mRNA levels of S100s were analyzed by Oncomine in different cancers. The expression levels of S100s were compared between clinical cancer specimens and normal controls by performing Student's *t*-test and assessing the *p* value. The fold change was set as 2 and the threshold of the *p* value was set as 0.01. The other parameters of Oncomine were set as the default settings.

### 2.2. Gene Expression Profiling Interactive Analysis (GEPIA) Dataset

GEPIA, available at http://gepia.cancer-pku.cn/ (March 11, 2020), is a database that provides diverse functions, including tumor and normal differential expression analysis, correlation analysis, profiling plotting, patient survival analysis, dimensionality reduction analysis, and the detection of similar genes based on different human tumor and normal samples from The Cancer Genome Atlas (TCGA) and Genotype-Tissue Expression (GTEx) programs [11]. The profiling, boxplot, and stage plot expressions of the S100 family genes were obtained from colon adenocarcinoma (COAD) and rectal adenocarcinoma (READ) tissue order via GEPIA, and the other default parameters of GEPIA were reserved.

The prognostic values of the mRNA expression of S100s were also evaluated by GEPIA. Patients with COAD and READ were split into two groups according to the median expression level (high vs. low expression) to analyze OS and disease-free survival (DFS). Patient samples were evaluated by the GEPIA survival plot, and the hazard ratio (HR) was presented with a 95% confidence interval (CI) and log *p* value.

### 2.3. cBioportal Analysis with TCGA Data

The TCGA database is a publicly funded project that includes high-throughput sequencing and pathological data of over 30 different human cancers [12]. cBioPortal (http://www.cbioportal.org/; accessed March 11, 2020) was applied to analyze S100s from the COAD (TCGA, Firehose Legacy) dataset including 379 cases. Mutations and putative CNAs from Genomic Identification of Significant Targets in Cancer (GISTIC) were selected as genomic profiles. Moreover, mRNA expression Z-scores relative to diploid samples (RNA Seq V2 RSEM) were chosen for the mRNA expression of genomic profiles, and protein expression Z-scores (RPPA) were selected for the protein/phosphoprotein expression levels. In addition, the top 20 coexpressed genes of the S100 family were also calculated for further analysis based on cBioPortal's online instructions according to the *p* value.

### 2.4. Integration of the Protein-Protein Interaction (PPI) Network and Module Analysis

The Search Tool for the Retrieval of Interacting Genes/Proteins (STRING, https://string-db.org/, version 11.0; accessed March 11, 2020) is an online database designed to predict PPI network information [13]. In the present study, the coexpression PPI network of the S100 family genes was analyzed via the STRING database, and 20 coexpressed genes with a combined score > 0.4 were relatively significantly altered. The PPI network of those genes mentioned above was constructed and visualized by the Cytoscape software (version 3.6.1), and the Molecular Complex Detection (MCODE) plug-in application of the software was used to find important modules for analysis with the criterion set as follows: k-core = 2, node score cut-off = 0.2, degree cutoff = 2, and maximum depth = 100.

### 2.5. Gene Ontology (GO) and Pathway Enrichment Analysis

GO analysis, covering the molecular function (MF), cellular component (CC), and biological process (BP) categories, is a commonly used method to study the characteristic attributes of large-scale genomic and transcriptome data [14]. Kyoto Encyclopedia of Genes and Genomes (KEGG) is a systematic collection of online web servers providing gene function and biological pathway information [15]. The Database for Annotation, Visualization, and Integrated Discovery (DAVID, https://david.ncifcrf.gov/; version 6.8; accessed March 11, 2020) is a free online bioinformatic resource that is designed to provide exhaustive functional annotation tools to identify enriched GO terms and visualize genes on KEGG pathway maps. The S100 family genes and their coexpressed genes of the top 5 modules in Cytoscape were input into the DAVID online tools to obtain the GO functions and KEGG pathways. Terms with a *p* value < 0.05 were considered statistically significant.

## 3. Results

### 3.1. Expression Levels of the S100 Gene Family in Patients with CRC

**Figure 1:**
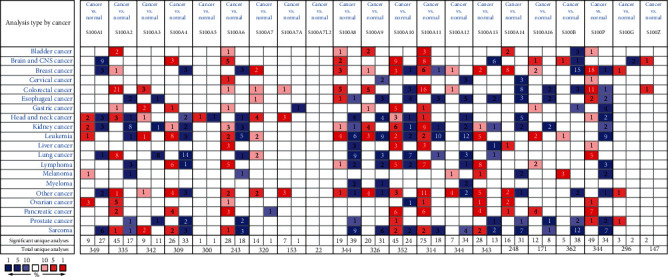
Expression levels of the S100 gene family in different types of cancers (Oncomine). The two colors of this graphic represent the upregulation (red) or downregulation (blue) of the S100 members.

We imported 21 genes in the S100 family reported by Anne et al. in the Oncomine database and compare their expression levels between the normal and cancerous CRC samples (see Figure 1). Using datasets from Oncomine, the mRNA expression levels of S100A2, S100A3, S100A6, S100A8, S100A9, S100A11, and S100P were significantly upregulated (a fold change of >1.5) in CRC tissues. Among various pathological types of CRC, two common types COAD and READ were selected for clarification. Results with Kaiser's dataset [16] showed that compared with normal samples, S100A2, S100A6, S100A8, S100A9, and S100P were overexpressed by 2.591, 2.037, 2.29, 1.951, and 4.911 folds in COAD tissues (see Table 1), and S100A6, S100A8, S100A9, and S100P were overexpressed by 2.735, 2.723, 2.097, and 4.879 folds in READ tissues. Results with Gaedcke's dataset [17] revealed that S100A2 was overexpressed by 9.983 folds in READ tissues. Analysis using Skrzypczak's dataset [18] showed that S100A2, S100A8, S100A9, and S100P were overexpressed by 5.846, 6.313, 3.941, and 3.212 folds in CRC tissues. Using Kaiser's dataset, the Oncomine analysis showed that S100A7A and S100A11 mRNA levels were upregulated by 1.875 and 1.734 folds in COAD tissues. And in READ patient, the two genes were overexpressed by 1.987 and 1.775 folds compared with normal samples. Using Skrzypczak's dataset, the analysis showed that S100A3 and S100A11 were overexpressed by 1.6 and 2.282 folds in COAD specimens, while S100A3 was overexpressed by 2.803 folds in READ specimens using Gaedcke's dataset (see Table 1).

Besides, S100A4, S100A7, S100A12, S100G, and S100Z were slightly overexpressed in the CRC datasets from Oncomine. S100A4 was overexpressed by 1.292 folds in COAD samples and 1.391 folds in READ samples using Kaiser's dataset and in CRC samples with a fold change of 1.73 using Skrzypczak's dataset. S100A7 was found in COAD with a fold change of 1.293 and in READ with a fold change of 1.27 using Kaiser's dataset and in CRC with a fold change of 1.411 using Skrzypczak's dataset. S100A12 was upregulated in CRC (fold change = 1.695) in Skrzypczak's dataset and COAD (fold change = 1.103) of Ki's dataset [19]. The mRNA level of S100G was slightly upregulated in colorectal adenocarcinoma samples (fold change = 1.025) in Skrzypczak's dataset, COAD samples (fold change = 1.19) of Notterman dataset [20], and READ samples (fold change = 1.025) using Gaedcke's dataset. The transcriptional levels of S100Z in COAD samples (fold change = 1.138) and READ samples (fold change = 1.115) slightly differed from those in the normal samples using Kaiser's dataset. Compared with normal samples, S100Z was similarly overexpressed by 1.059 folds in CRC specimens using Skrzypczak's dataset. The expression levels of S100A1, S100A5, and S100A13 were similar between CRC and normal tissues (see Table 1).

By contrary, the mRNA levels of S100A14 and S100A16 were significantly downregulated (a fold change of >1.5) using CRC datasets from Oncomine. Using Kaiser's dataset, the Oncomine analysis showed that compared with normal samples, S100A14 was downexpressed by 2.18 and 2.143 folds in COAD and READ tissues (see Table 1), and S100A16 was downexpressed by 1.573 and 1.549 folds in COAD and READ tissues, respectively. The S100A10 mRNA level was marginally downregulated by 1.075 and 1.107 folds in COAD and READ tissues using Kaiser's dataset. Using Skrzypczak's dataset, the analysis revealed that S100A10 was also downexpressed by 1.169 folds in COAD specimens and that the S100B level was downregulated by 1.065 fold in CRC tissues compared with normal tissue controls (see Table 1).

### 3.2. Relationships between the Expressions of S100 Family Genes and Pathological Types of CRC

**Figure 2:**
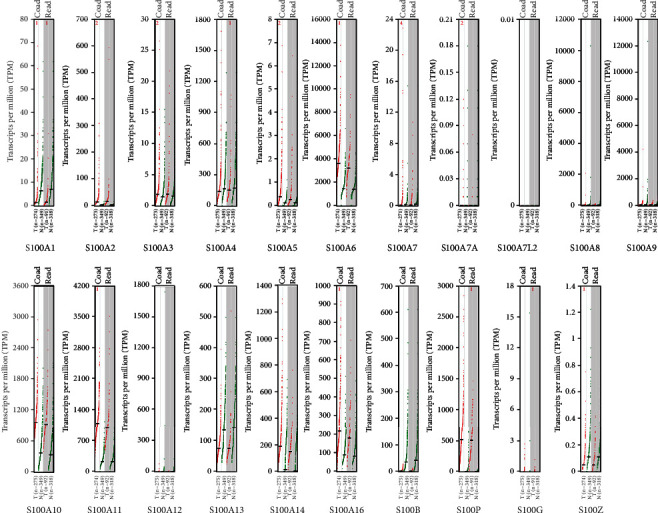
The transcriptional expression of the S100 family members in COAD and READ. Red indicates expression in tumor tissues, and green indicates expression in corresponding normal tissues.

**Figure 3:**
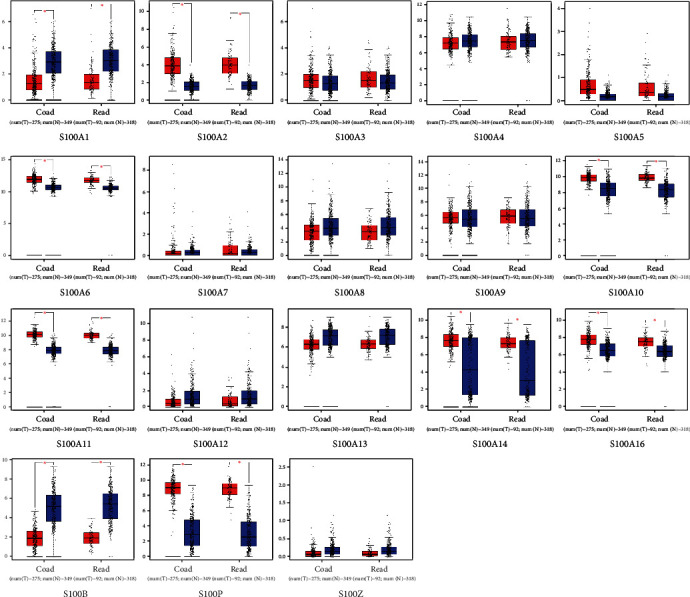
The expression of S100 family members of CRC patients. Red indicates expression in tumor tissues, and blue indicates expression in normal colon tissues. Significantly expressed genes are listed with an asterisk (^∗^*p* < 0.05).

**Figure 4:**
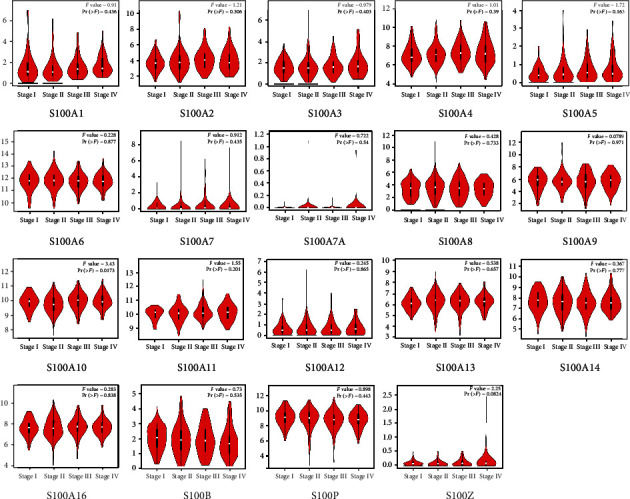
Expression of the S100 family members in CRC patients with various clinical stages.

The analysis of S100 gene expressions in CRC and normal colon tissues was conducted by using mRNA data obtained from the GEPIA database. The results showed that the expression levels of S100A2, S100A3, S100A5, S100A6, S100A7, S100A10, S100A11, S100A14, S100A16, S100P, and S100G were higher in COAD and READ tissues than those in normal controls (see Figure 2) and that S100A2, S100A6, S100A10, S100A11, S100A14, S100A16, and S100P expressions were significantly higher in CRC specimens (see Figure 3). However, S100A1, S100A4, S100A8, S100A9, S100A12, S100A13, S100B, and S100Z were downexpressed in COAD when compared with READ (see Figure 2), of which S100A1 and S100B expression levels were particularly lower than the levels of the other S100 genes in CRC tissues (see Figure 3). However, the detection of several genes (e.g., S100A7A, S100A7L2, and S100G) using GEPIA was unavailable due to insufficient data. The expression levels of the S100 family genes were also analyzed in COAD and READ at different stages. Only the S100A10 subgroup showed significant differences in expression levels between different stages (see Figure 4).

### 3.3. Survival Analysis of the S100 Gene Family in Patients with CRC by GEPIA

**Figure 5:**
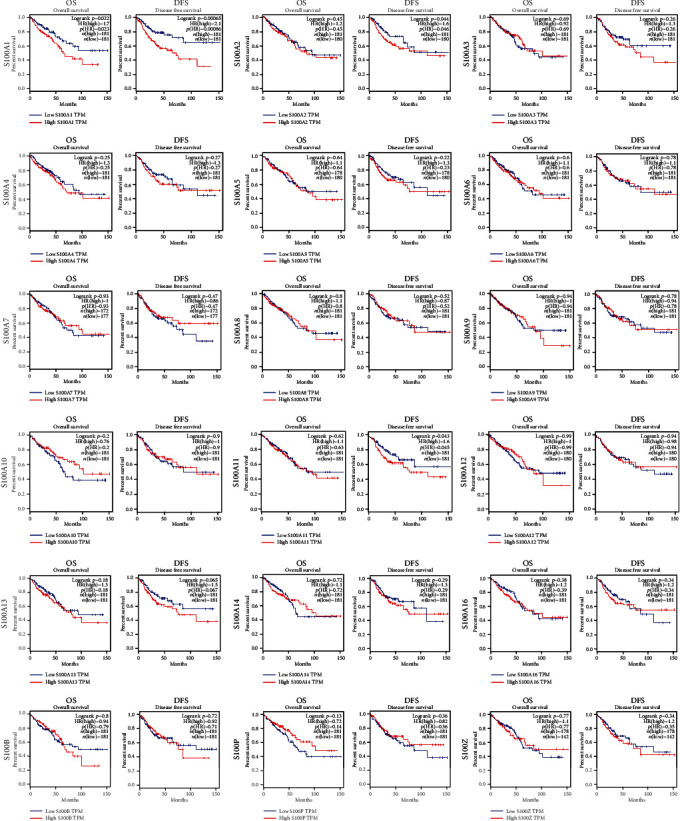
Survival analysis of S100 gene family in patients with CRC (GEPIA).

Survival-associated S100 genes were identified by GEPIA database. Despite unavailable analysis for the correlations between S100A7A, S100A7L2, and S100G expressions and OS or DFS of CRC patients due to insufficient data, the analysis for the other S100 genes revealed that the S100A1 overexpression was associated with worse OS of patients with COAD and READ (*p* < 0.05) (see Figure 5) and that S100A2~S100A14, S100A16, S100B, S100P, and S100Z expressions had no significant correlations with OS of the patients. The S100A4 overexpression was correlated with poor OS of the patients, while elevated S100A10 and S100P levels were correlated with favorable OS. It was also found that increased S100A1, S100A2, and S100A11 mRNA levels were apparently associated with poor DFS of COAD patients (see Figure 5). An elevated S100A13 level was seemingly associated with worse DFS of COAD and READ patients (*p* = 0.065), but with a nonsignificant difference. Overexpressed S100A3 and S100Z were associated with poor DFS, while S100A7 and S100P overexpressions were associated with favorable DFS of COAD and READ patients. The other genes of the S100 family had no clear correlations with DFS in CRC.

### 3.4. The Correlations between S100 Family Genes in Patients with COAD

**Figure 6:**
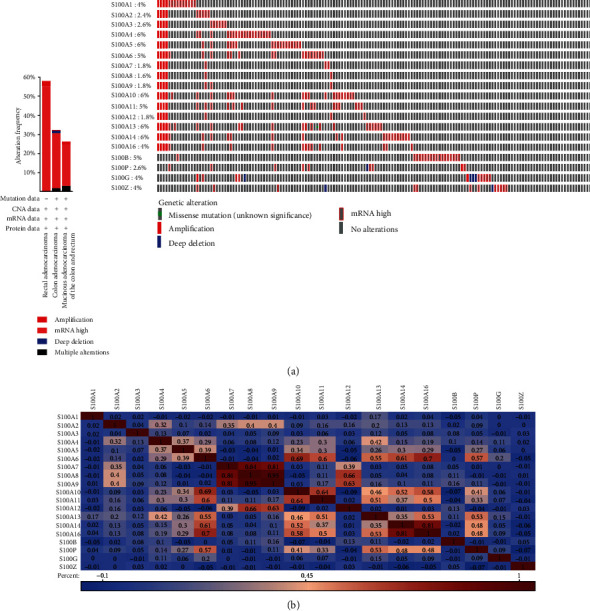
S100 gene expression and mutation analysis in COAD (cBioPortal). (a) Cancer type summary and oncoprint of the S100 members in cBioPortal. (b) Heatmap of the correlation among S100 members. Pearson correlation coefficients are exhibited as a color gradient from blue (negative correlation) to pink to reddish brown (positive correlation).

We used the cBioPortal online tool to analyze correlations among altered S100 family genes in COAD specimens and pinpointed 126 specimens (126/379, 33%) showing abnormally expressed S100 genes related to COAD, of which 31 to 69 samples exhibited two or more abnormally expressed S100 genes (see Figure 6(a)). The Pearson correlation between these S100 genes in COAD specimens was also calculated by analyzing their mRNA expression data (RNA Seq V2 RSEM) from TCGA database (Firehose Legacy) using the cBioPortal platform. The results revealed significantly positive associations between the following pairs of S100 genes: S100A4 with S100A13; S100A6 with S100A10, S100A11, S100A13, S100A14, S100A16, and S100P; S100A7 with S100A8 and S100A9; S100A8 with S100A7, S100A9, and S100A12; S100A9 with S100A7, S100A8, and S100A12; S100A10 with S100A6, S100A11, S100A13, S100A14, S100A16, and S100P; S100A11 with S100A6, S100A10, S100A13, and S100A16; S100A12 with S100A8 and S100A9; S100A13 with S100A4, S100A6, S100A10, S100A11, S100A16, and S100P; S100A14 with S100A6, S100A10, S100A16, and S100P; S100A16 with S100A6, S100A10, S100A11, S100A13, S100A14, and S100P; and S100P with S100A6, S100A10, S100A13, S100A14, and S100A16. Their coexpression networks were depicted in Figure 6(b).

### 3.5. PPI Network and Module Analysis of S100 Proteins and Their Coexpressed Genes

**Figure 7:**
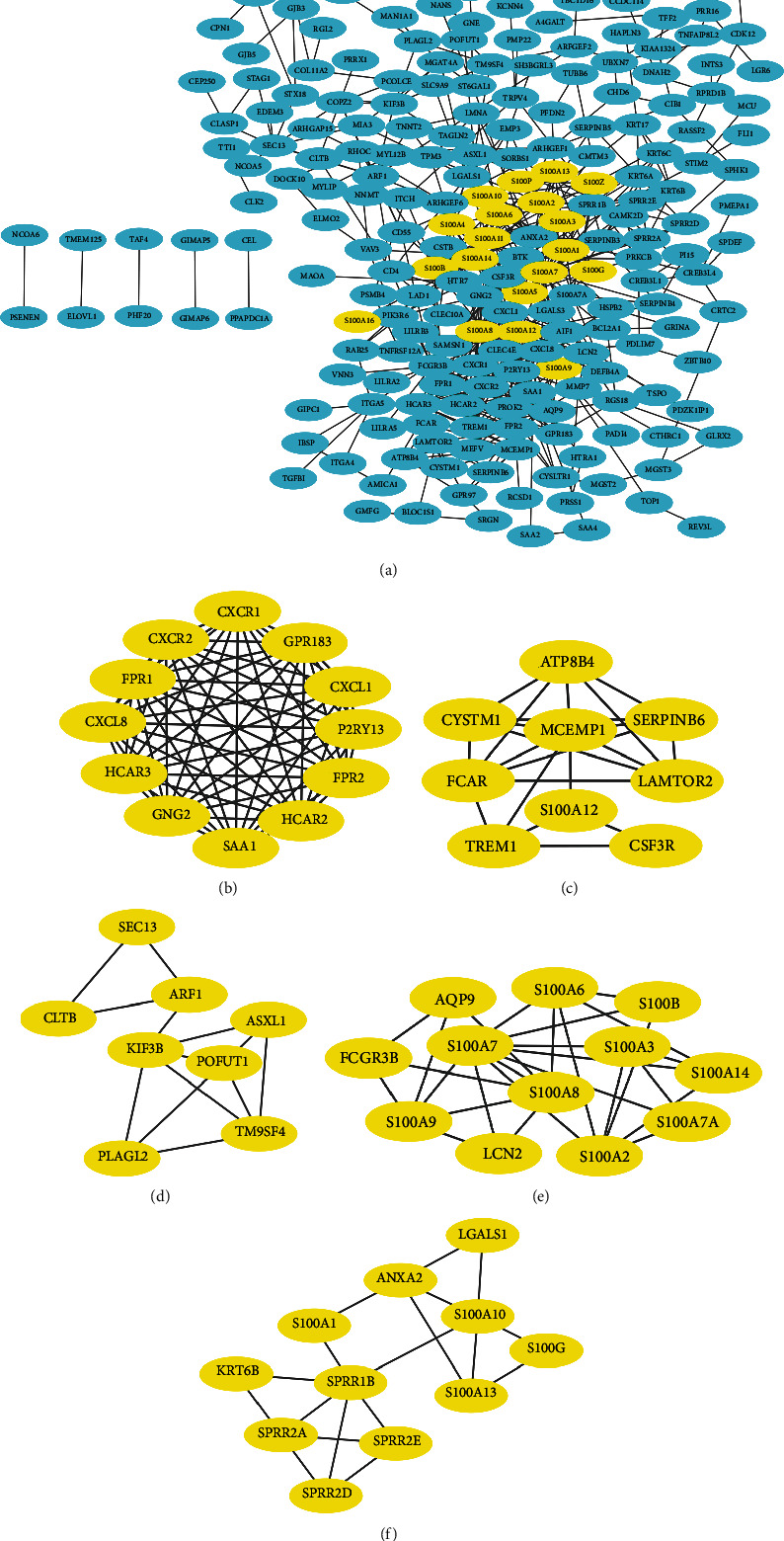
PPI network constructed by STRING and module composition visualized by Cytoscape. (a) Visualization the PPI network of S100 members and their mutation-related genes. S100 members are circled in yellow. (b) Module 1 of the top 5 modules with maximum depth of 100, node score cut-off of 0.2, k-core of 2, and degree cutoff of 2. (c) Module 2 of the top 5 modules. (d) Module 4 of the top 5 modules. (e) Module 3 of the top 5 modules. (f) Module 5 of the top 5 modules.

We then constructed a coexpressed gene cnetwork of the S100 family linked to CRC based on 20 most relevant genes. The PPI network of those genes mentioned above was obtained from STRING, and the results were visualized by the Cytoscape software. A network of 212 nodes and 574 edges was constructed, and 281 genes were analyzed through the MCODE plug-in (see Figure 7(a)). Additionally, the top 5 modules with default parameters were selected to elucidate the interactions between S100 proteins and other molecules. Finally, MCODE analysis generated 5 modules as follows: Module 1 with 12 nodes and 66 edges (see Figure 7(b)), Module 2 with 9 nodes and 21 edges (see Figure 7(c)), Module 4 with 8 nodes and 13 edges (see Figure 7(d)), Module 3 with 12 nodes and 27 edges (see Figure 7(e)), and Module 5 with 11 nodes and 18 edges. S100 family genes were mostly distributed among the top 5 modules.

### 3.6. GO Function and KEGG Pathway Enrichment Analyses of CRC-Related S100 Genes

**Figure 8:**
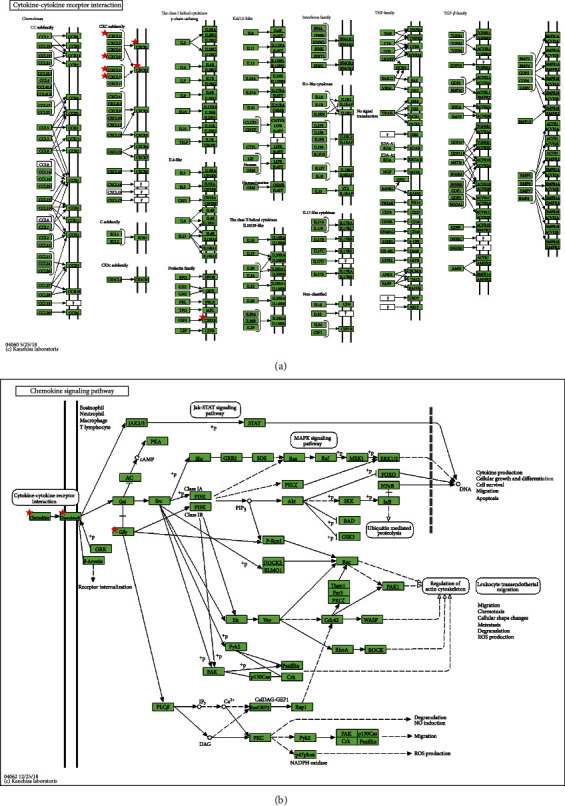
KEGG pathway enrichment of the S100 genes and their significantly related genes selected from the top 5 module analysis. (a) Cytokine-cytokine receptor interaction. (b) Chemokine signaling pathway.

The enriched GO functions of S100 family genes and the 5 modules of coexpressed genes were analyzed by DAVID online database for the CC, MF, and BP categories. As shown in Table 2, the top 6 GO terms of CC for these coexpressed genes consisted of extracellular exosomes, extracellular regions, the cornified envelope, the plasma membrane, the perinuclear region of cytoplasm, and an integral component of the plasma membrane. The top 6 GO terms of MF involved RAGE receptor binding, calcium ion binding, S100 protein binding, calcium-dependent protein binding, interleukin-8 binding, and Toll-like receptor 4 binding. The top 6 GO terms of BP included inflammatory responses, chemotaxis, peptide cross-linking, chemokine-mediated signaling pathways, G-protein coupled receptor signaling pathways, and positive regulation of cytosolic calcium ion concentration. The KEGG pathway enrichment analyses (see Table 3) showed that S100 alterations and the coexpressed genes that altered frequently were particularly enriched in the chemokine signaling pathways (*p* = 0.001459) and cytokine-cytokine receptor interaction (*p* = 0.003873) (see Figure 8).

## 4. Discussion

Many reports have documented that S100 gene dysregulation is related to several cancers [3, 7, 8, 10, 21]. Although the roles of S100 genes in tumorigenesis and prognosis of human cancers have been partly confirmed [2, 9, 10, 21], further extensive bioinformatics analyses of the S100 family in CRC have not yet been performed. This study for the first time reports the prognostic (DFS, OS) values of the S100 gene family in CRC using bioinformatics tools, and our findings will underpin further studies on the mechanisms of dysregulated S100 genes in CRC, therapeutic targets, and optimization of treatment plans with improved prognosis. As analyses for S100A7A, S100A7L2, and S100G expressions in CRC are unavailable due to a lack of data, we merely focus on other members of the S100 family that are obviously related to the progression of CRC.

In our study, gene expression analyses show that S100A2, S100A11, and S100P expression levels in CRC tissues are significantly higher than those in noncancerous tissues, and S100A3 and S100A9 mRNAs are highly expressed in cancer tissues compared with normal tissue controls. By contrary, S100B is significantly downexpressed in CRC tissues. However, Oncomine analysis and GEPIA have yielded inconsistent results of S100A8, S100A10, S100A14, and S100A16 expression levels. Based on the Oncomine database, there are no obvious distinctions in S100A1, S100A4, S100A5, S100A6, and S100A13 expressions between cancer tissues and normal colon mucosa, and S100A7, S100A12, and S100Z are slightly overexpressed in CRC tissues. Whereas, GEPIA shows that S100A5 and S100A6 are overexpressed in COAD and READ tissues, and S100A1, S100A4, S100A7, S100A12, S100A13, and S100Z expression levels are downregulated in cancerous tissues.

As for the prognostic value of dysregulated S100 genes, our results show a significant correlation between S100A10 and CRC at different stages of progression (*p* = 0.0173). However, such a strong correlation has not been observed in any other member of the S100 family. Notably, the elevated S100A1 level is significantly correlated with both poor OS and DFS of CRC patients. The S100A2 overexpression is correlated with worse DFS of patients, but its predictive value for poor OS cannot be confirmed. The S100A11 overexpression only indicates worse DFS of CRC patients. An elevated S100A13 level has a nonsignificant association with worse DFS, with a considerable trend toward significance (*p* = 0.065).

Since the expression levels of the S100 genes are not completely parallel in the two databases, we mainly focused on prognostic S100 members that are consistent in gene expression levels. S100A1 proteins are abundantly expressed in the central neuronal system, heart muscle, and skeletal muscle [22]. Although S100A1 is proved to be a biomarker in human cancers, its role in colon cancers has been rarely been studied. S100A1 protein expressions are marginally higher in the colon connective tissues of normal samples and adenoma with low-grade dysplasia than CRC tissues and high-grade dysplastic lesions [23]. Bronckart et al. report the presence of S100A1 expression in node-negative colon cancer and S100A1 deficiency in node-positive colon cancer [23]. This indicates that S100A1 can be a candidate biomarker for the prognosis of early-stage colon cancer.

S100A2 gene expressions in colon cancers have also been reported [24] and are associated with poor OS and DFS of CRC patients [25, 26]. The high mRNA expression of S100A2 is associated with poor relapse-free survival, suggesting that S100A2 can be an independent risk factor for the recurrence of advanced CRC patients [27]. However, S100A2 as a predictor of stage progression in CRC has not been proven.

S100A3 plays an important role in tumorigenesis and progression of a variety of human cancers [28–30]. Activated and overexpressed S100A3 is associated with tumorigenesis, tumor occurrence, and progression of CRC [31], and S100A3 may be a potential target for CRC treatment. Consistently, our finding showed that the S100A3 overexpression predicted poor DFS of CRC patients (*p* = 0.26).

S100A8 and S100A9 which are mainly expressed in myeloid cells naturally form a stable heterodimer and involve in inflammatory processes that lead to autoimmune diseases and many human cancers [32, 33]. S100A8 and S100A9 have been proposed as crucial proinflammatory factors and contribute to premetastatic niche formation in CRC, which are consistent with our finding that S100A8 and S100A9 show inflammatory chemotactic effects in CRC. Kim et al. reveal that S100A8/9 heterocomplexes are upregulated in colon cancers and promote tumor progression [34]. However, the heterocomplex shows nonsignificant prognostic values in CRC in our study.

S100A10 intracellularly colocalizes with annexin A2 and involves in the translocation of S100A10 to the cytosolic face of the plasma membrane [35]. Zhang et al. report that S100A10 is correlated with cellular invasiveness, angiogenesis, and metastasis of CRC cells [31, 36]. Shang et al. find that S100A10 overexpressions in CRC can enhance oxaliplatin (L-OHP) sensitivity [37, 38], which is consistent with our results that S100A10 overexpressions significantly associate with longer OS of CRC patients. S100A11 is located in the cytoplasm of tumor cells and highly expressed in CRC tissues compared with adjacent normal tissues. This suggests that S100A11 involves in the cellular growth of progressive CRC [39, 40]. S100A13 is considered to be a potent angiogenic biomarker for astrocytic gliomas and melanoma, but its role in CRC is rarely reported [41, 42].

S100B alone can significantly increase proliferation and angiogenesis in intestinal colon cancer Caco-2 cells, which is considered to be an “ideal bridge” linking colonic inflammation and cancer [43]. Seguella et al. show that S100B markedly increases cell proliferation and invasiveness in CRC cells. Moreover, overexpressed S100B is implicated in postoperative relapse and a poor prognosis in CRC [44]. In our study, though S100B is significantly downexpressed in CRC tissues, contrary to our expectation, S100B supression has no associations with stage progression, OS or DFS in CRC.

Emoto et al. first identified S100P as a new calcium-modulated protein in the human placenta in 2001 [1]. Previous evidences support that S100P protein and mRNA expressions in cancerous tissues significantly increase compared with normal colon mucosa tissues [45]. Wang et al. report that stage I-III CRC patients with positive S100P protein expressions exhibited shorter OS compared with negative S100P expressions. However, in our research, patients having higher S100P levels show an overall trend of better OS and DFS, without significant differences.

Besides, limitations in our study must be acknowledged. First, as differences between samples and data resources are inevitable, same genes that are inconsistent expressed in the two databases may result in cognitive confusion. Second, the gene expression analyses are performed based on online databases, which means our findings must be verified in more large-sample clinical trials on CRC.

## 5. Conclusion

In summary, we have systematically analyzed expressions of 21 genes in the S100 family and explored their prognostic value in CRC by using the Oncomine and GEPIA databases, STRING, Cytoscape, cBioportal, and the DAVID database. Among the 21 S100 genes, 3 (S100A1, S100A2, and S100A11) are significantly associated with the prognosis of CRC patients, and only S100A10 is significantly correlated with CRC stage and progression, suggesting that S100A1, S100A2, and S100A11 can serve as potential prognostic markers. Therefore, the prognostic value of the S100 family, especially S100A10, needs to be verified in animal experiments and clinical trials. Our study will underpin researches on molecular mechanisms of S100 proteins and relevant signaling pathways in CRC progression.

Our research offers novel insights into the contribution of the S100 family to the prognosis and progression of CRC and paves a way for new S100-targeted therapies for CRC.

## Figures and Tables

**Table 1:** The changes in the expression of the S100 family genes at the transcription level between different types of colorectal cancer and colon tissues (Oncomine database).

	Types of colorectal cancer vs. normal colon	Fold change	*p* value	*t*-test	Ref
S100A1	Rectal adenocarcinoma vs. normal	1.009	0.457	0.111	Kaiser Colon [25]
	Colon adenocarcinoma vs. normal	1.003	0.484	0.043	Kaiser Colon [25]
	Colorectal adenocarcinoma vs. normal	1.042	0.143	1.077	Skrzypczak Colorectal [27]
S100A2	Colorectal carcinoma vs. Normal	5.846	2.66E-13	9.915	Skrzypczak Colorectal [27]
	Colon adenocarcinoma vs. normal	2.591	4.94E-07	7.205	Kaiser Colon [25]
	Rectal adenocarcinoma vs. normal	9.983	5.77E-46	24.76	Gaedcke Colorectal [26]
S100A3	Colorectal carcinoma vs. normal	1.6	1.82E-08	6.425	Skrzypczak Colorectal [27]
	Rectal adenocarcinoma vs. normal	2.803	4.20E-24	14.11	Gaedcke Colorectal [26]
S100A4	Colorectal carcinoma vs. normal	1.73	1.18E-04	3.923	Skrzypczak Colorectal [27]
	Colon adenocarcinoma vs. normal	1.292	8.00E-02	1.52	Kaiser Colon [25]
	Rectal adenocarcinoma vs. normal	1.391	1.00E-01	1.365	Kaiser Colon [25]
S100A5	Colorectal carcinoma vs. normal	1.066	7.00E-02	1.498	Skrzypczak Colorectal [27]
	Colon adenocarcinoma vs. normal	1.09	2.70E-02	2.212	Kaiser Colon [25]
	Rectal adenocarcinoma vs. normal	1.057	1.57E-01	1.055	Kaiser Colon [25]
S100A6	Colon adenocarcinoma vs. normal	2.037	6.88E-04	4.931	Kaiser Colon [25]
	Rectal adenocarcinoma vs. normal	2.735	5.54E-04	4.409	Kaiser Colon [25]
S100A7	Colorectal carcinoma vs. normal	1.411	6.00E-03	2.637	Skrzypczak Colorectal [27]
	Colon adenocarcinoma vs. normal	1.293	3.52E-04	3.777	Kaiser Colon [25]
	Rectal adenocarcinoma vs. normal	1.27	5.00E-03	3.11	Kaiser Colon [25]
S100A7A	Colon adenocarcinoma vs. normal	1.875	2.70E-04	7.056	Kaiser Colon [25]
	Rectal adenocarcinoma vs. normal	1.987	1.25E-04	5.321	Kaiser Colon [25]
S100A2L2	NA	NA	NA	NA	NA
S100A8	Colorectal carcinoma vs. normal	6.313	5.37E-06	5.126	Skrzypczak Colorectal [27]
	Colon adenocarcinoma vs. normal	2.29	3.89E-04	4.478	Kaiser Colon [25]
	Rectal adenocarcinoma vs. normal	2.723	1.80E-02	2.488	Kaiser Colon [25]
S100A9	Colorectal carcinoma vs. normal	3.941	1.14E-08	6.784	Skrzypczak Colorectal [27]
	Colon adenocarcinoma vs. normal	1.951	6.05E-04	5.139	Kaiser Colon [25]
	Rectal adenocarcinoma vs. normal	2.097	1.80E-02	2.455	Kaiser Colon [25]
S100A10	Colorectal adenocarcinoma vs. normal	-1.169	9.97E-01	-2.95	Skrzypczak Colorectal [27]
	Colon adenocarcinoma vs. normal	-1.075	7.60E-01	-0.719	Kaiser Colon [25]
	Rectal adenocarcinoma vs. normal	-1.107	6.66E-01	-0.444	Kaiser Colon [25]
S100A11	Colorectal adenocarcinoma vs. normal	2.282	2.21E-11	10.392	Skrzypczak Colorectal [27]
	Colon adenocarcinoma vs. normal	1.734	4.00E-03	4.425	Kaiser Colon [25]
	Rectal adenocarcinoma vs. normal	1.775	3.00E-03	4.487	Kaiser Colon [25]
S100A12	Colorectal carcinoma vs. normal	1.695	4.00E-03	2.743	Skrzypczak Colorectal [27]
	Colon adenocarcinoma vs. normal	1.103	1.97E-01	0.856	Ki Colon [28]
S100A13	Colorectal carcinoma vs. normal	1.199	4.60E-02	1.721	Skrzypczak Colorectal [27]
	Colon adenocarcinoma vs. normal	1.133	1.51E-01	1.102	Kaiser Colon [25]
	Rectal adenocarcinoma vs. normal	1.081	3.13E-01	0.501	Kaiser Colon [25]
S100A14	Colorectal carcinoma vs. normal	-1.655	1.00E+00	-3.731	Skrzypczak Colorectal [27]
	Colon adenocarcinoma vs. normal	-2.18	1.00E+00	-8.84	Kaiser Colon [25]
	Rectal adenocarcinoma vs. normal	-2.143	1.00E+00	-5.957	Kaiser Colon [25]
S100A16	Colon adenocarcinoma vs. normal	-1.573	9.92E-01	-3.348	Kaiser Colon [25]
	Rectal adenocarcinoma vs. normal	-1.549	9.91E-01	-3.084	Kaiser Colon [25]
S100B	Colorectal carcinoma vs. normal	-1.065	6.46E-01	-0.378	Skrzypczak Colorectal [27]
S100P	Colorectal carcinoma vs. normal	3.212	1.91E-06	5.587	Skrzypczak Colorectal [27]
	Colon adenocarcinoma vs. normal	4.911	3.00E-03	4.798	Kaiser Colon [25]
	Rectal adenocarcinoma vs. normal	4.879	2.00E-03	4.233	Kaiser Colon [25]
S100G	Colorectal adenocarcinoma vs. normal	1.025	2.08E-01	0.821	Skrzypczak Colorectal [27]
	Colon adenocarcinoma vs. normal	1.19	1.20E-01	1.202	Notterman Colon [29]
	Rectal adenocarcinoma vs. normal	1.025	1.10E-02	2.325	Gaedcke Colorectal [26]
S100Z	Colorectal carcinoma vs. normal	1.059	4.50E-02	1.738	Skrzypczak Colorectal [27]
	Colon adenocarcinoma vs. normal	1.138	0.081	1.698	Kaiser Colon [25]
	Rectal adenocarcinoma vs. normal	1.115	1.13E-01	1.398	Kaiser Colon [25]

**Table 2:** Gene Ontology analysis of the S100 genes and their most significantly coexpressed genes in CRC.

Category	Term	Count	%	*p* value	FDR
GOTERM_BP_DIRECT	GO:0006954~inflammatory response	9	17.30769	9.11E-06	0.012361
GOTERM_BP_DIRECT	GO:0006935~chemotaxis	6	11.53846	2.24E-05	0.030396
GOTERM_BP_DIRECT	GO:0018149~peptide cross-linking	4	7.692308	0.000367	0.497246
GOTERM_BP_DIRECT	GO:0070098~chemokine-mediated signaling pathway	4	7.692308	0.001028	1.385837
GOTERM_BP_DIRECT	GO:0007186~G-protein coupled receptor signaling pathway	9	17.30769	0.003201	4.257847
GOTERM_BP_DIRECT	GO:0007204~positive regulation of cytosolic calcium ion concentration	4	7.692308	0.006236	8.138288
GOTERM_CC_DIRECT	GO:0070062~extracellular exosome	22	42.30769	5.91E-06	0.006523
GOTERM_CC_DIRECT	GO:0001533~cornified envelope	4	7.692308	0.000271	0.299178
GOTERM_CC_DIRECT	GO:0005886~plasma membrane	23	44.23077	0.000633	0.696457
GOTERM_CC_DIRECT	GO:0005576~extracellular region	12	23.07692	0.003667	3.973017
GOTERM_CC_DIRECT	GO:0048471~perinuclear region of cytoplasm	6	11.53846	0.027144	26.19097
GOTERM_CC_DIRECT	GO:0005887~integral component of plasma membrane	9	17.30769	0.037498	34.41178
GOTERM_MF_DIRECT	GO:0050786~RAGE receptor binding	7	13.46154	1.99E-13	2.24E-10
GOTERM_MF_DIRECT	GO:0005509~calcium ion binding	15	28.84615	9.3E-09	1.05E-05
GOTERM_MF_DIRECT	GO:0044548~S100 protein binding	4	7.692308	6.44E-06	0.007239
GOTERM_MF_DIRECT	GO:0048306~calcium-dependent protein binding	4	7.692308	0.000634	0.710203
GOTERM_MF_DIRECT	GO:0019959~interleukin-8 binding	2	3.846154	0.008683	9.337967
GOTERM_MF_DIRECT	GO:0035662~Toll-like receptor 4 binding	2	3.846154	0.011561	12.253

**Table 3:** KEGG pathway analysis of the S100 genes and their related coexpressed genes in colorectal cancer.

Category	Term	Count	%	*p* value	FDR
KEGG_PATHWAY	hsa04062: chemokine signaling pathway	5	9.615385	0.001459	1.441856
KEGG_PATHWAY	hsa04060: cytokine-cytokine receptor interaction	5	9.615385	0.003873	3.787008

## Data Availability

The data used to support the findings of this study are available from the corresponding author upon request. The data could also obtain from following open online websaites, Oncomine (www.oncomine.org), GEPIA, available at http://gepia.cancer-pku.cn/ (March 11, 2020), cBioPortal (http://www.cbioportal.org/; accessed March 11, 2020), The Search Tool for the Retrieval of Interacting Genes/Proteins (STRING, https://string-db.org/, version 11.0; accessed March 11, 2020) and The Database for Annotation, Visualization and Integrated Discovery (DAVID, https://david.ncifcrf.gov/; version 6.8; accessed March 11, 2020).
